# Remote sensing for site selection in vegetation survey along a successional gradient in post‐industrial vegetation

**DOI:** 10.1002/ece3.70200

**Published:** 2024-08-27

**Authors:** Quadri A. Anibaba, Marcin K. Dyderski, Gabriela Woźniak, Andrzej M. Jagodziński

**Affiliations:** ^1^ Institute of Dendrology Polish Academy of Sciences Kórnik Poland; ^2^ Institute of Biology, Biotechnology and Environmental Protection, Faculty of Natural Sciences University of Silesia Katowice Poland

**Keywords:** functional diversity, indicator species, phylogenetic diversity, post‐mining sites, species composition

## Abstract

Vegetation characteristics are an important proxy to measure the outcome of ecological restoration and monitor vegetation changes. Similarly, the classification of remotely sensed images is a prerequisite for many field ecological studies. We have a limited understanding of how the remote sensing approach can be utilized to classify spontaneous vegetation in post‐industrial spoil heaps that dominate urban areas. We aimed to assess whether an objective a priori classification of vegetation using remotely sensed data allows for ecologically interpretable division. We hypothesized that remote sensing‐based vegetation clusters will differ in alpha diversity, species, and functional composition; thereby providing ecologically interpretable division of study sites for further analyses. We acquired remote‐sensing data from Sentinel 2A for each studied heap from July to September 2020. We recorded vascular plant species and their abundance across 400 plots on a post‐coal mine in Upper Silesia, Poland. We assessed differences in alpha diversity indices and community‐weighted means (CWMs) among remote sensing‐based vegetation units. Analysis of remotely sensed characteristics revealed five clusters that reflected transition in vegetation across successional gradients. Analysis of species composition showed that the 1st (early‐succession), 3rd (late‐succession), and 5th (mid‐succession) clusters had 13, 10, and 12 exclusive indicator species, respectively, however, the 2nd and 4th clusters had only one species. While the 1st, 2nd, and 4th can be combined into a single cluster (early‐succession), we found the lowest species richness in the 3rd cluster (late‐succession) and the highest in the 5th cluster (mid‐succession). Shannon's diversity index revealed a similar trend. In contrast, the 3rd cluster (late‐succession) had significantly higher phylogenetic diversity. The 3rd cluster (late‐succession) had the lowest functional richness and the highest functional dispersion. Our approach underscored the significance of a priori classification of vegetation using remote sensing for vegetation surveys. It also highlighted differences between vegetation types along a successional gradient in post‐mining spoil heaps.

## Graphical abstract

We assess whether objective a priori classification of vegetation using remotely sensed data allows for ecologically interpretable division. We found that remote sense features differentiated early‐, mid‐, and late‐successional stages, and species richness and diversity followed an arch‐shaped pattern along this successional gradient. Species composition revealed indicative species specific to the successional stage, and remote sensing allows low‐cost selection of study sites to assess restoration success.

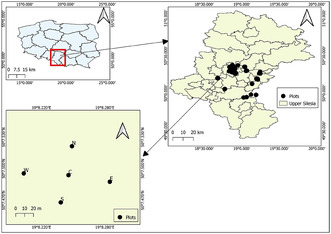

## 1 INTRODUCTION

Ecological succession, a sequence of changes in communities over time, is a widely studied process of community ecology (Connell & Slatyer, 1977; Li et al., 2023; Meiners et al., 2015; Prach & Walker, 2020; Pulsford et al., 2016). Across ecosystems, there is evidence of changes in community composition and traits over succession (Fu et al., 2023; Hobbs et al., 2007; Nowak et al., 2022; Pickett et al., 1987; Rejmanek & Katwyk, 2005). Yet there is doubt about the mechanisms responsible for this change (Backhaus et al., 2021). Theory predicts that the colonization probability as assumed by the lottery model and environmental filtering is responsible for community assembly in the early stage of succession, while at the late successional stage, interspecific competition is assumed to be the major driving force (Foster et al., 2004; Pacala & Tilman, 1994; Purschke et al., 2013).

Earlier studies of plant community composition along successional gradients in post‐mining areas have majorly focused on temporal changes in species (taxonomic) composition (Alday et al., 2011a; Frouz et al., 2008; Prach et al., 2001; Rehounková & Prach, 2006; Wiegleb & Felinks, 2001b), less frequently on changes in single traits or functional groups (Prach et al., 1997; Rehounková & Prach, 2006; Woźniak et al., 2011). However, approaches based solely on species composition do not account for the species ecological differences. Exploring functional groups cannot permit the possibility that species within functional groups may be functionally distinct from one another (Anibaba et al., 2023; Marquard et al., 2009). Moreover, these studies use a phytosociological approach for vegetation classification and plot locality selection, which is a disadvantage since vegetation patterns in novel ecosystems such as post‐mining areas are distributed in a complex mosaic and most vegetation is heterogenous (Woźniak et al., 2021). Therefore, there is a need to explore the potential of remote sensing for vegetation classification of post‐mining areas.

Remote sensing is a good tool for the detection of land‐use changes (e.g., Akiwumi & Butler, 2008), vegetation health (e.g., Erener, 2011), and vegetation type, as well as for assessing topographic features and vegetation height (Wężyk et al., 2015). The latter may be simply used for tree biomass estimations (Badreldin & Sanchez‐Azofeifa, 2015; Jagodziński et al., 2018). In recent years numerous studies used remote sensing for the detailed characterization of ecosystem species composition and diversity, as well as their transformation under global environmental changes (e.g., Cârlan et al., 2020; Martin‐Gallego et al., 2020; Tymińska‐Czabańska et al., 2022; Unberath et al., 2019). Especially, advancement in high‐resolution satellites and sensors has enabled increases in the scope and accuracy of estimations (e.g., Große‐Stoltenberg et al., 2018; Hawryło et al., 2020; Silva et al., 2021).

In contrast to natural ecosystems, fewer studies used remote sensing to investigate post‐mining vegetation. For example, Game et al. (1982) used remotely sensed data to analyze changes in space and time among patches of vegetation on a surface coal mine undergoing natural succession in Missouri, U.S.A. They classified vegetation development on spoil heaps into three stages representing vegetation cover categories. Schmidt and Glaesser (1998) used remote sensing data to monitor the environmental impact of open‐cast lignite mining in Eastern Germany. They classified the vegetation into two clusters: bare open‐cast areas and areas of less dense vegetation. Other studies have described the applicability of remote sensing for the recognition of land‐use types and coarse vegetation categories (e.g., LeClerc & Wiersma, 2017; Maimaitijiang et al., 2015; Small, 2001). Yang et al. (2018) used remote sensing for the assessment of vegetation disturbance and recovery in surface mining sites. However, only a few studies have used remote sensing to detect more detailed vegetation units such as those that can be described based on species composition and functional traits (Woźniak et al., 2021). Most studies identified a priori‐defined vegetation clusters, assessing the accuracy of supervised classification. However, none of them assessed whether using only remotely sensed data can result in vegetation classification into ecologically interpretable units. Here we filled this gap by using a remote sensing approach to inform both the classification and ground data collection of vegetation for the assessment of taxonomic, functional, and phylogenetic diversity. Our study can help provide a method for detailed ecological research.

The vegetation classification of novel ecosystems is important for the ecological restoration of degraded land (Hobbs et al., 2006; Kowarik, 2011). It is necessary to assess large areas of land both before, and after reclamation. For the effective management of post‐industrial land to fulfill the required social functions and other ecosystem services, there is a need to make a simple and relatively cheap inventory of its basic features. The novelty of our study is an analysis of the chronosequence of spontaneously developed vegetation, where we might assume either confirmation or rejection of remote sensing applicability for mapping vegetation. We expect that similarly to other types of ecosystems successfully recognized using airborne methods (e.g., Akiwumi & Butler, 2008; Hoffmann et al., 2019; Wężyk et al., 2015), post‐industrial spontaneous vegetation can also be remotely sensed. Due to the high convergence of vegetation types and habitat filtering (e.g., Prach et al., 2017), we may also expect weak relationships between plant species composition and remotely sensed features. Therefore we aimed to assess whether objective a priori classification of vegetation using remotely sensed data allows for ecologically interpretable division. We hypothesized that remote sensing‐based vegetation clusters will differ in alpha diversity, species, and functional composition, providing an ecologically interpretable division of study sites for further analyses.

## 2 METHODS

### 2.1 Study area

We conducted our study in Upper Silesia, a region with a long tradition of coal mining (since the 18th century). Long‐lasting mining activity resulted in large amounts of post‐industrial sites, occupying more than 2000 ha (Szczepańska, 1987). These objects shape the anthropogenic landscape, built of Carboniferous sediments on pre‐Cambrian crystalline rocks. The Carboniferous mudstone and sandstone complexes are mixed with numerous coal elements. They are also overlain by Triassic carbonate formations (Cabała et al., 2004). Coal mine spoil heaps are habitats that are difficult for plant development. They are characterized by extreme abiotic conditions, for example, large variations in humidity and daily temperatures (often reaching 50°C), high salinity, lack of soil, susceptibility to erosion, substrate instability, dusting, chemical, and thermal activity, and also biotic parameters such as lack of a seed bank and a deficiency of nutrients in the substrate (Woźniak et al., 2021). These habitat features impact vegetation able to colonize these post‐industrial sites (Bradshaw, 2000; Prach et al., 2013; Woźniak, 2010).

From the list of 112 post‐mining objects with available information about age, size, vegetation, and reclamation method (Woźniak, 2010) we excluded 31 sites that have been underrepresented, for example, thermally active, regularly formed, or spoil heaps outlying in terms of size or age. Within the remaining 81 post‐mining objects, we randomly selected 60 objects (75% of post‐mining objects), proportionally to size, age, and characteristics of the surrounding landscapes.

### 2.2 Remote sensing data acquisition

For each spoil heap, we downloaded harmonized Sentinel 2A satellite images using the “sen2r” package (Ranghetti et al., 2020). We used the same package for the selection and correction of images. We selected images with <10% cloud cover and acquired them between June and September 2020. We used images from 1st July, 9th September, and 14th September. All images have a pixel size of 10 m (best available spatial resolution). Although we excluded pixels with thick clouds, some thin clouds could affect our calculations of spectral indices. According to timeanddate.com archival weather forecasts, on July 1, 2020 the Katowice sky was moderately cloudy, while on 9th and 14th September it was cloudless. For each date, we obtained raw bands and calculated spectral indices (Table 1). We decided to use raw bands and spectral indices that are correlated with plant chemical composition (Gamon et al., 1997; Hoffmann et al., 2019; Merzlyak et al., 1999) and both vegetation intensity and biomass (Boelman et al., 2003; Butterfield & Malmström, 2009). These variables in the previous study (Woźniak et al., 2021) supported the classification of vegetation on post‐mining sites. We also included burn indices (García & Caselles, 1991; Trigg & Flasse, 2001; Vermote et al., 2016) that discriminate areas with black and gray surfaces, typical of early successional stages, where remnants of hard coal and rocks are visible on the surface. We used the “rgugik” package (Dyba & Nowosad, 2021) to obtain a digital elevation model and digital surface model for each site. We used the difference between them as an approximation of vegetation height (Table 1).

**TABLE 1:** List of remotely sensed variables.

Abbreviation	Name, source	Explanation
AVI	Ashburn Vegetation Index (Ashburn, 1978)	SB850 – SB655; correlated with biomass
EVI	Enhanced Vegetation Index (Huete et al., 2002)	$2.5*\frac{\mathrm{SB}850-\mathrm{SB}655}{\left(\mathrm{SB}850+6*\mathrm{SB}655-7.5*\mathrm{SB}460\right)-1}$, correlated with vegetation cover and biomass
MIRBI	*Mid‐Infrared Burn Index* (Trigg & Flasse, 2001)	10*SB2180‐9.8*SB1580 + 2, useful in the detection of burned areas and assessing burn severity; here we used it for the classification of nonvegetated sites
NBR	*Normalized Difference NIR/SWIR Normalized Burn Ratio* (García & Caselles, 1991)	$\;\frac{\left(\mathrm{SB}850-\mathrm{SB}1580\right)}{\left(\mathrm{SB}850+\mathrm{SB}1850\right)},$ another index for burn detection, but based on other bands WIR
NBR2	*Normalized Burn Ratio 2* (Trigg & Flasse, 2001; Vermote et al., 2016)	$\frac{\left(\mathrm{SB}1580-\mathrm{SB}2180\right)}{\left(\mathrm{SB}1580+\mathrm{SB}2180\right)},$ burn index based on short‐wave infrared
NDVI	Normalized Difference Vegetation Index (Rouse et al., 1974)	$\frac{\left(\mathrm{SB}850-\mathrm{SB}655\right)}{\left(\mathrm{SB}850+\mathrm{SB}655\right)},$ classic index of vegetation, correlated with biomass and cover
SB460	Reflectance of 460 nm band	Blue reflectance. The reflectance of particular bands is useful for the construction of other spectral indices and classifications
SB555	Reflectance of 555 nm band	Green, see above
SB655	Reflectance of 655 nm band	Red, see above
SB850	Reflectance of 850 nm band	Visible and near‐infrared, see above
SB1580	Reflectance of 1580 nm band	Short‐wave infrared, see above
SB2180	Reflectance of 2180 nm band	Short‐wave infrared, see above
VH	Vegetation height	Difference between the digital elevation model and the digital surface model from LIDAR measurements, expressed in meters

### 2.3 Remote sensing data processing

We assessed the optimal number of clusters using an elbow method: we ran k‐means classification for 2:20 clusters and then we compared decreases in total within‐cluster sum of squares. An optimal number of clusters was reached when a further decrease in the sum of squares (when adding another cluster) was not significant. After ensuring that five is the optimal number, we ran a k‐means algorithm and assigned each map pixel to one of five classes. Assessed vegetation on remote‐sensing‐based clusters was formed over time and was not spatially consistent (i.e., some clusters are younger than others) (see Figure S1 in Supplementary material for the age distribution of remote‐sensing‐based clusters). We used Principal components analysis (PCA) to explore differences in remotely sensed parameters of a particular cluster. Before PCA we scaled predictors to overcome differences in their ranges. We developed PCA using the “vegan” package (Oksanen et al., 2018).

### 2.4 Field site selection and data collection

**FIGURE 1:**
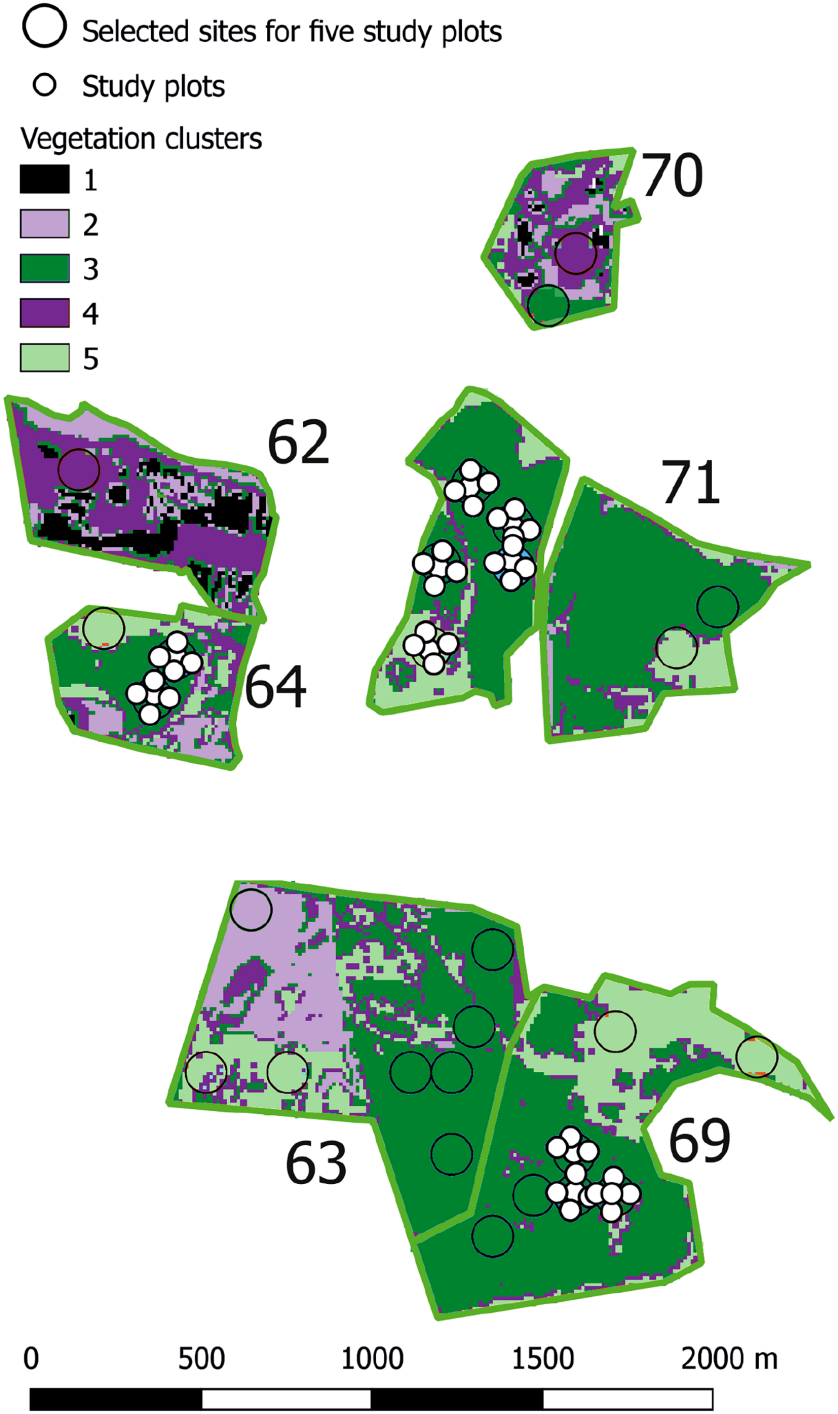
Example of site and plot selection using the background of remotely sensed classes and their distribution on chosen post‐industrial objects (black numbers).

**FIGURE 2:**
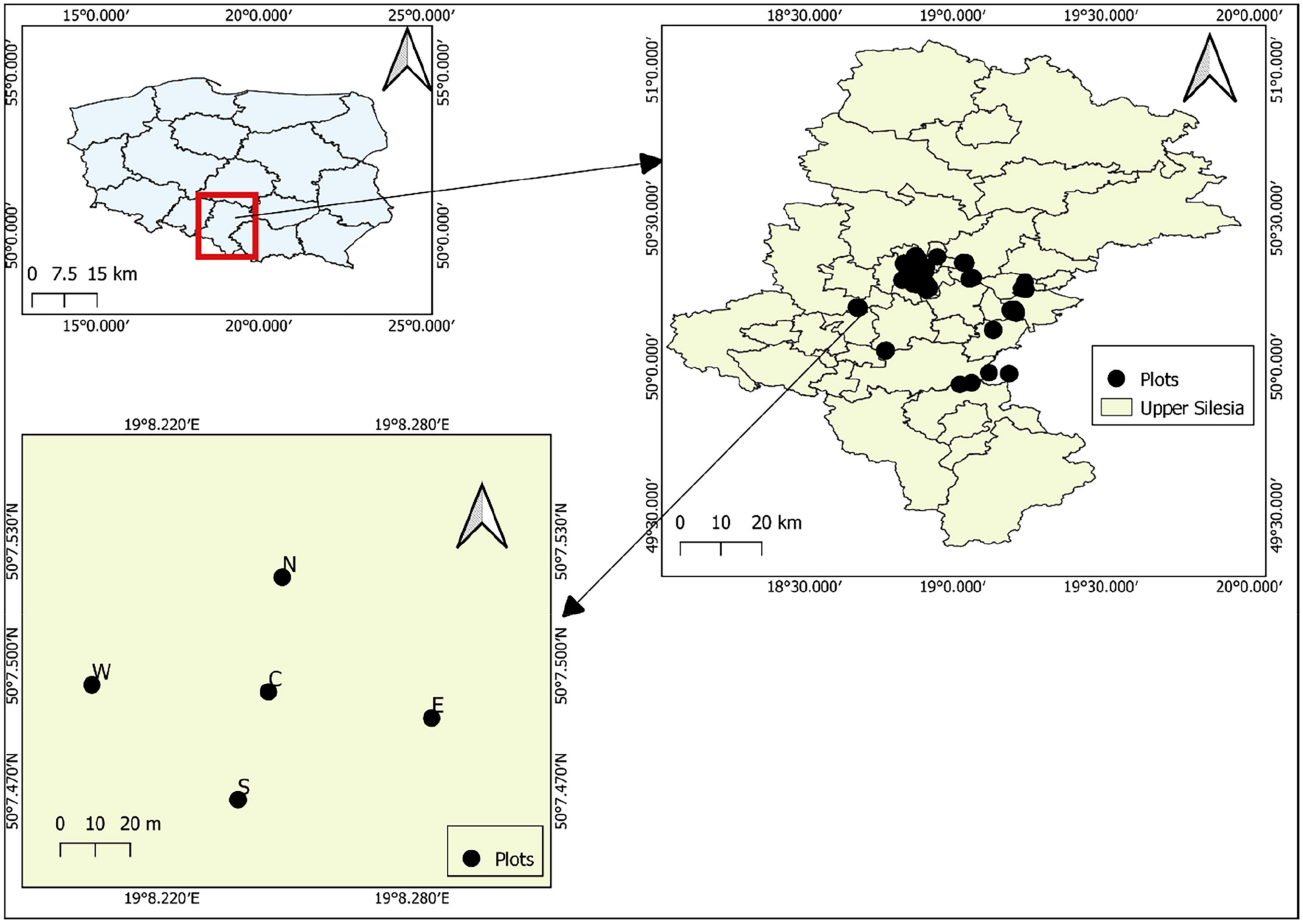
Distribution of study plots (*n* = 400) in Upper Silesia. The study design shows plots in the north (N), south (S), east (E), and west (W) directions at 50 m from the central plot (C).

After assigning each pixel to one of the five clusters, we polygonized the raster layer using the sf package (Pebesma, 2018) in R and selected patches with an area of more than 14,400 m^2^, that is, large enough to host five plots in a block, with distances of 50 m from each other (Figure 1). To find potential locations of study sites, we used a regular grid (120 × 120 m) and at the grid intersections, we drew circles (*r* = 60 m). For further analyses, we used only homogenous circles (with a minimum variance of class number < 0.2). That way we selected 551 potential sites that can host a block of five study plots. Then, we manually inspected their airborne images in Google Earth Pro, to exclude sites with roads, buildings, or water bodies. After exclusion, we obtained 427 potential sites. Within each spoil heap and cluster, we randomly assigned numbers of potential sites, that reflected priority of plot establishment. We then establish 400 study plots (i.e., 80 sites) proportionally to cluster abundance: in cluster 1 – four sites, cluster 2–19 sites, cluster 3–19 sites, cluster 4–14 sites, and cluster 5–24 sites. For site selection, first, we assigned random numbers to each potential site within each heap, within each cluster. Then, we used these numbers in priority order for selecting sites during fieldwork. In the summer of 2021, we visited each heap and firstly we went to the site with the number one in each cluster. If we confirmed in the field that the site is suitable for establishing study plots (homogeneity, lack of major human interventions, not intersected by roads, etc.), then we established five plots there. If it was impossible to establish a study plot we visited the next number. Although we primarily intended to establish one site per cluster per heap, due to dynamic changes in heaps ownership structure and construction work that started before fieldwork, it was impossible and we had to exclude some heaps from the study and establish more sites per cluster in some heaps. In total, we established 80 sites that were planned (i.e., 400 study plots) (Figure 2). Still, due to the inaccessibility of some heaps, we changed the structure of cluster proportions: three in cluster 1, 19 in cluster 2, 17 in cluster 3, 15 in cluster 4, and 26 in cluster 5. Within each plot, we recorded vascular plant species and their cover using the Londo scale (Londo, 1976). The size of the study plot (28.3 m^2^) is large enough to describe the species composition of nonforest vegetation and typical of usually used plots in sampling synanthropic, meadow, and grassland vegetation. Chytrý and Otýpková (2003) recommend even smaller study plots (16 m^2^) for such vegetation types.

### 2.5 Vegetation characteristics

We aimed to check whether the obtained clusters differ in species composition, functional composition, and biodiversity. For that reason, we decided to assess species composition, the functional composition based on trait values, and alpha diversity indices. To obtain that, we prepared a dataset of species abundances, traits, and phylogeny. For the set of vascular plant species present in study plots, we obtained a phylogenetic tree derived from the mega tree included in the “V.phylo.maker” package (Jin & Qian, 2022). We also acquired functional traits (Table 2) from LEDA (Kleyer et al., 2008), BIEN (Maitner et al., 2018), BiolFlor (Klotz et al., 2002), and Pladias (Chytrý et al., 2021) databases, and ecological indicator values from Ellenberg and Leuschner (2010). We imputed missing data using the random forest‐based imputation (Penone et al., 2014), implemented in the *missForest* package (Stekhoven & Bühlmann, 2012). We developed a model based on known trait values and phylogenetic eigenvectors (Diniz‐Filho et al., 1998), obtained using the *PVR* package (Santos, 2018). The first 15 phylogenetic eigenvectors covered 59.3% of the variation in phylogenetic distances among species. The normalized root mean squared error of imputed traits was 1.011 for continuous predictors and the proportion of falsely classified categorical variables was 0.079.

**TABLE 2:** Traits used in the study, their ranges, variation coefficient (CV), and completeness.

Numeric traits	Min	Max	Mean	CV [%]	Completeness [%]
EIV‐Light (EIV‐L)	1	9	6.7	22.2	98.1
EIV‐Moisture (EIV‐M)	2	11	5.3	33.7	86.8
EIV‐Soil reaction (EIV‐SR)	1	9	6.2	28.8	69.1
EIV‐Nutrients (EIV‐N)	1	9	5.0	42.7	87.7
EIV‐Temperature (EIV‐T)	2	8	5.6	16.5	72.9
Flowering beginning [months]	1	9	5.5	21.6	98.9
Flowering duration [months]	1	12	3.4	40.7	98.9
Specific leaf area (SLA) [cm^2^ g^−1^]	51.8	899.1	247.7	45.5	87.9
Leaf dry mass content (LDMC) [mg g^−1^]	0.3	509.5	230.6	33.7	81.1
Seed mass (SM) [mg]	0.001	13737.6	103.6	941.3	94.5
Maximum height (H) [m]	0.033	60.0	4.6	230.5	98.9

Categorical traits	Number of classes	Classes and their frequency	Completeness [%]
Life form	8	Chamaephytes (4.4%), Geophytes (7.1%), Hemicryptophytes (51.8%), Hydrophytes (0.82%), Lianas (3.3%), Phanerophytes (16.7%), Therophytes (15.3%)	100.0
Pollination mode—insect	2	Yes (71.7%), no (28.3%)	95.9
Pollination mode—selfing	2	Yes (52.0%), no (48.0%)	95.9
Pollination mode—wind	2	Yes (31.1%), no (68.9%)	95.9

We described alpha diversity for each plot using six indices. For taxonomic diversity, we calculated species richness and Shannon's diversity index. For phylogenetic diversity, we used Faith's phylogenetic diversity (PD; that is, the sum of phylogenetic tree branch lengths, representing all species present in the community) and mean pairwise phylogenetic distance (MPD). For functional diversity, we measured the functional richness (FRic) expressing the quantity of plant functional types present in a community and functional dispersion (FDis), expressing the size of community species traits hypervolume within the functional trait space (Laliberté & Legendre, 2010; Mason et al., 2005).

We used a null model approach to test whether the phylogenetic diversities differed from the randomly generated assemblage of species. We calculated PD and MPD using the “PhyloMeasures” package (Tsirogiannis & Sandel, 2016) while FRic and FDis using the “FD” package (Laliberté et al., 2014).

### 2.6 Data analysis

We analyzed data using R software v. 4.0.1 (R Core Team, 2021). We assessed the species composition of study plots (using the presence–absence transformed data) by nonmetric multidimensional scaling (NMDS), implemented in the “vegan” package (Oksanen et al., 2018). Before NMDS we excluded 28 plots without vegetation and 14 plots with only one species that produced artifacts and did not allow NMDS to reach a convergence (producing *n* = 400–28–14 = 358 for NMDS). Using the IndVal method (Cáceres & Legendre, 2009) we assessed whether a species is an indicator of a spoil heap cluster (i.e., a particular species is more frequent/associated with a particular cluster than others). For each species, we provided IndVal statistics and *p*‐value informing about the strength and significance of the association (Cáceres & Legendre, 2009).

We assessed differences in alpha diversity indices and CWMs using linear mixed‐effects models (LMMs), accounting for dependencies among plots within blocks and heaps by the random intercepts. For species richness, we used generalized LMM (GLMM) assuming Poisson distribution. We used the “lme4” (Bates et al., 2015) and “lmerTest” (Kuznetsova et al., 2017) packages to develop LMMs and GLMM. Before GLMM development we checked potential problems with zero‐inflation and overdispersion using tests implemented in the “DHARMa” package (Hartig, 2020). We assessed the impacts of remote sensing‐based clusters on response variables by ANOVA. Although models could reveal differences among forest types with *p*‐values < .05, via Tukey's *posteriori* tests we applied a single‐step adjustment of *p*‐values, to account for multiple hypothesis testing. Single‐step adjustment decreases the probability of committing Type I error (i.e., rejection of the true null hypothesis), and also accounts for correlations among variables tested (Bretz et al., 2011). We also reported Akaike's Information Criterion (AIC) for full and null (intercept and random effects only, AIC_0_) models, to show how including clusters increases the model performance. We calculated marginal ($R_{m}^{2}$) and conditional ($R_{c}^{2}$) coefficients of determination, indicating the proportion of variability explained by fixed effects only and by both random and fixed effects, respectively (Nakagawa & Schielzeth, 2013) using the “MuMIn” package (Bartoń, 2017).

## 3 RESULTS

### 3.1 Remotely sensed characteristics of vegetation

**FIGURE 3:**
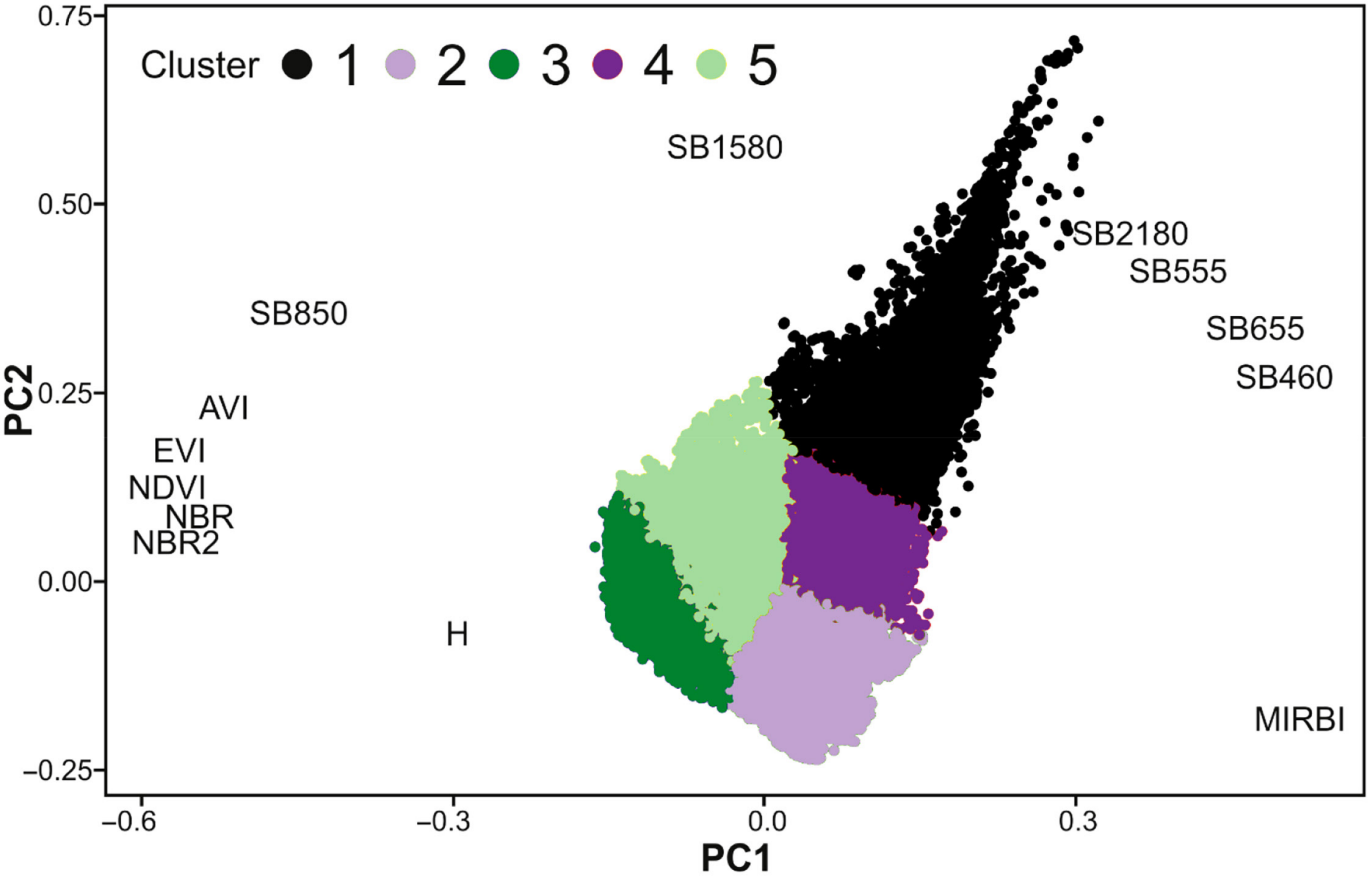
Result of principal components analysis (PCA) of remotely sensed characteristics of pixels (Table 1), colored according to k‐means clustering.

Analysis of remotely sensed characteristics of clusters revealed that the PC1 axis explained 62.4% of variability while PC2 explained 25.2% (Figure 3). Axis PC1 divided vegetated sites (i.e., clusters 3 and 5) from sparsely vegetated sites (i.e., clusters 1, 2, and 4). This axis of division reflected both spectral vegetation and burn indices, as well as vegetation height. The latter is divided between cluster 3, covered by woody vegetation and cluster 5, covered by herbaceous vegetation. Axis PC2 differentiated sites with high values of infrared reflectance (SB1580 and SB2180), from sites with lower reflectance of these bands. This axis divided less‐vegetated sites into three clusters, according to thermal activity and proportion of reflectance. Cluster 2 had low reflectance of all bands, indicating close to black surface color.

### 3.2 Species and functional composition

**FIGURE 4:**
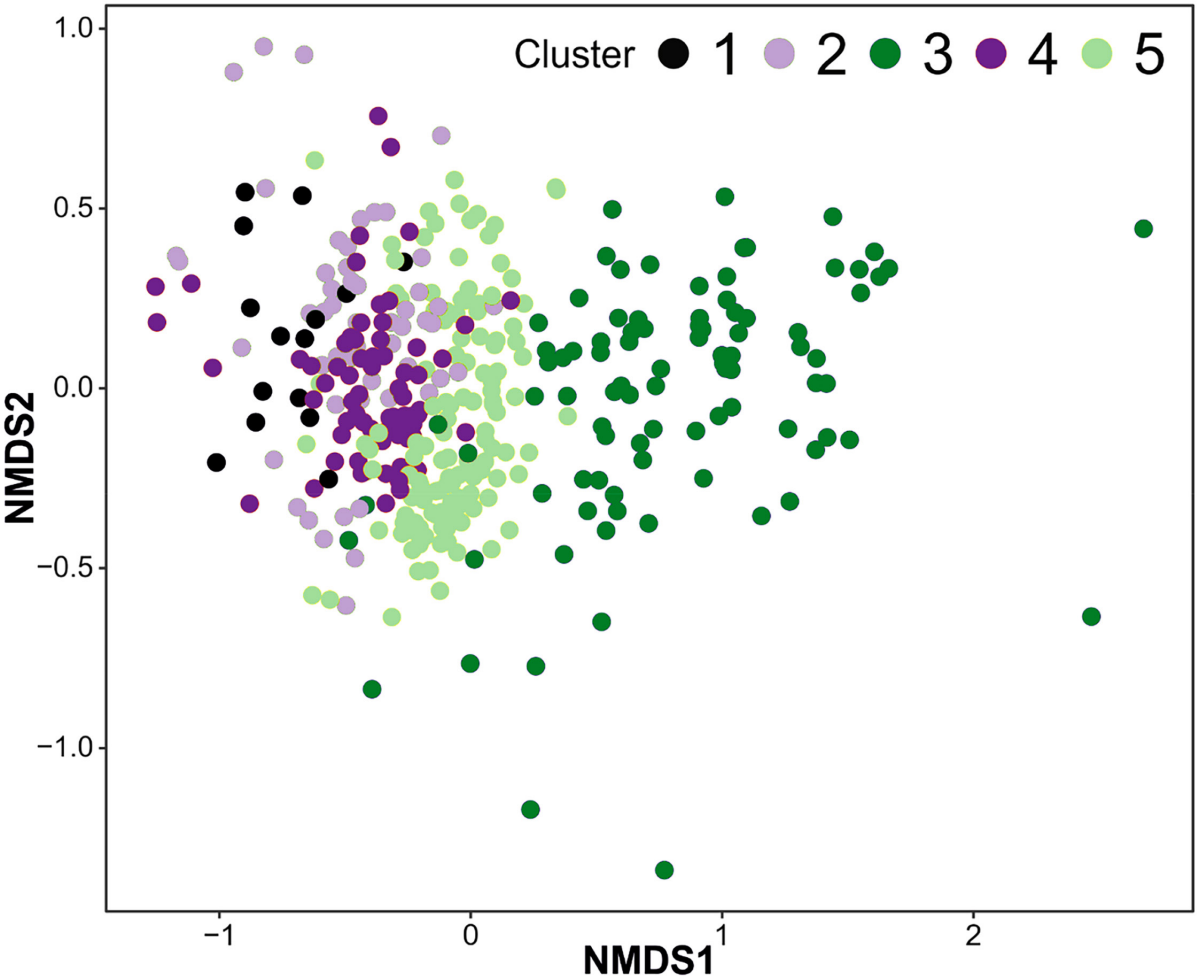
Result of nonmetric multidimensional scaling (NMDS, stress = 0.1631) of vegetation in study plots (points), colored according to k‐means clustering (Figure 2).

In total, we observed vascular plants within 372 plots, 15 in the 1st cluster, 62 in the 2nd cluster, 93 in the 3rd cluster, 72 in the 4th cluster, and 130 in the 5th cluster. We found more than one species of vascular plants in 358 plots. Ordination (NMDS) revealed differentiation of remote sensing‐based clusters along main axes (Figure 4). Main axis (NMDS1) differentiated 1st, 2nd, and 4th clusters from 3rd and 5th. However, these three left clusters (1st, 2nd, and 4th) representing initial vegetation did not differentiate in ordination space. The most distinct was 3rd cluster, representing woody vegetation, which had almost no overlap with other clusters.

**FIGURE 5:**
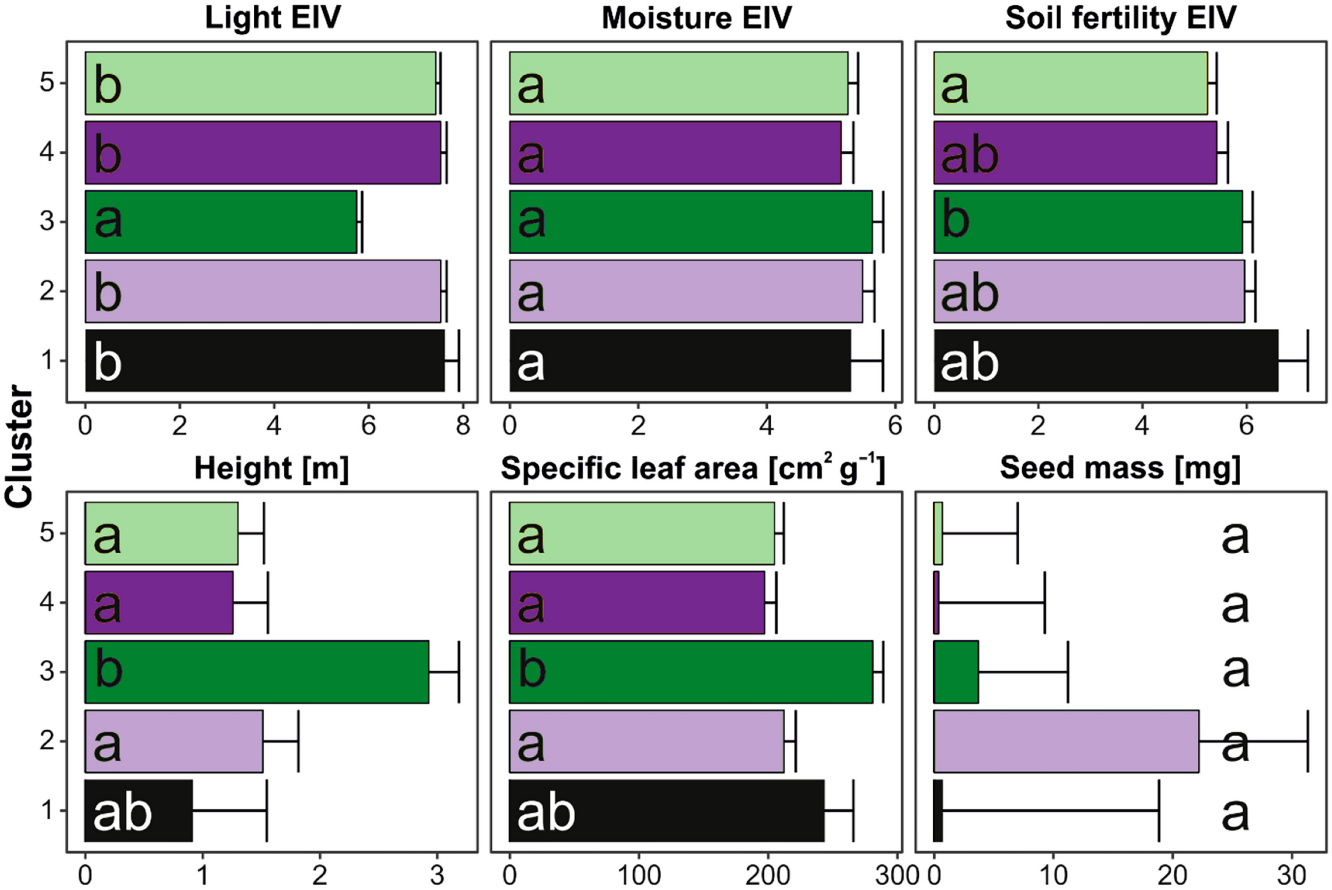
Mean (+SE) values of the community‐weighted mean (CWM) of ecological indicator values (EIV) and functional traits describing studied vegetation among remote sensing‐based clusters, assessed using linear mixed‐effects models (Table 4). The same letters denote groups that did not differ at the confidence level *α* = .05 after multiple hypotheses adjustment, according to a Tukey *posteriori* test.

Analysis of species composition revealed that the 1st, 3rd, and 5th clusters had 13, 10, and 12 exclusively indicative species, respectively, while the 2nd and 4th—had only one species (*Epilobium parviflorum* and *Carlina vulgaris*, respectively). The first cluster differed from others by a high frequency of the grassland species *Lolium perenne*, and ruderals: *Oxybasis* spp., *Chenopodium album*, and *Echinochloa crus‐galli* (Table 3). The third cluster differed by a high frequency of forest species: *Lolium giganteum*, *Circaea lutetiana*, *Millium effusum*, and *Poa nemoralis*, as well as forest‐edge species: *Rubus idaeus*, *Geum urbanum*, and *Impatiens parviflora*. The fifth cluster differed by a high frequency of meadow and grassland species: *Achillea millefolium*, *Dactylis glomerata*, *Festuca rubra*, *Galium mollugo*, *Silene latifolia*, and *Vicia cracca*. The 3rd cluster had the lowest number of indicative species in common with other clusters. Analysis of functional composition revealed that the most distinct 3rd cluster differed significantly from other clusters in light and soil fertility EIVs, and height and SLA CWMs (Table 4; Figure 5). However, cluster 5 (second most distinct in NMDS) did not differ from other clusters in any CWMs.

**TABLE 3:** The frequency (%) of plant species within remote sensing‐based clusters (1–5; Figure 1) and the strength of association assessed using the IndVal method (we bolded frequency for clusters where species is indicative).

Species	1	2	3	4	5	IndVal statistic	*p*‐Value
*Matricaria discoidea*	**13.3**	0.0	0.0	0.0	0.0	0.365	0.002
*Bromus sterilis*	**13.3**	0.0	0.0	1.4	0.0	0.359	0.003
*Amaranthus retroflexus*	**13.3**	0.0	0.0	2.8	0.0	0.332	0.010
*Epilobium roseum*	**13.3**	4.8	0.0	0.0	3.8	0.266	0.034
*Setaria helvola*	**20.0**	0.0	0.0	0.0	0.0	0.447	0.001
*Sonchus asper*	**20.0**	9.7	0.0	0.0	0.0	0.407	0.002
*Plantago major*	**33.3**	4.8	0.0	5.6	6.2	0.485	0.001
*Cirsium palustre*	**33.3**	12.9	0.0	13.9	3.1	0.385	0.010
*Echinochloa crus‐galli*	**46.7**	8.1	0.0	12.5	6.9	0.440	0.003
*Chenopodium album*	**53.3**	17.7	0.0	4.2	0.8	0.649	0.001
*Oxybasis rubra*	**60.0**	16.1	0.0	2.8	0.0	0.584	0.001
*Oxybasis glauca*	**66.7**	8.1	0.0	0.0	5.4	0.750	0.001
*Lolium perenne*	**80.0**	0.0	0.0	0.0	3.1	0.877	0.001
*Epilobium parviflorum*	0.0	**17.7**	2.2	0.0	0.0	0.404	0.003
*Lolium giganteum*	0.0	0.0	**10.8**	0.0	0.0	0.328	0.009
*Clematis vitalba*	0.0	0.0	**11.8**	0.0	0.0	0.344	0.005
*Prunus serotina*	0.0	0.0	**12.9**	0.0	0.0	0.359	0.008
*Rubus idaeus*	0.0	0.0	**12.9**	0.0	0.0	0.359	0.008
*Circaea lutetiana*	0.0	0.0	**19.4**	0.0	0.0	0.440	0.002
*Poa nemoralis*	0.0	0.0	**19.4**	0.0	0.0	0.440	0.002
*Sambucus nigra*	0.0	0.0	**19.4**	0.0	0.0	0.440	0.001
*Milium effusum*	0.0	0.0	**20.4**	0.0	0.0	0.452	0.003
*Impatiens parviflora*	0.0	0.0	**43.0**	0.0	0.0	0.656	0.001
*Geum urbanum*	0.0	0.0	**57.0**	0.0	1.5	0.754	0.001
*Carlina vulgaris*	0.0	3.2	0.0	**12.5**	2.3	0.290	0.039
*Scabiosa ochroleuca*	0.0	0.0	0.0	0.0	**11.5**	0.340	0.013
*Thymus pulegioides*	0.0	0.0	0.0	0.0	**12.3**	0.351	0.011
*Arrhenatherum elatius*	0.0	1.6	0.0	0.0	**12.3**	0.349	0.011
*Festuca pratensis*	0.0	0.0	0.0	0.0	**13.8**	0.372	0.008
*Lysimachia vulgaris*	0.0	0.0	0.0	0.0	**14.6**	0.382	0.008
*Medicago falcata*	0.0	0.0	0.0	1.4	**16.9**	0.407	0.005
*Vicia cracca*	0.0	0.0	1.1	0.0	**19.2**	0.433	0.001
*Silene latifolia*	0.0	1.6	1.1	0.0	**19.2**	0.422	0.003
*Galium mollugo*	0.0	0.0	0.0	0.0	**25.4**	0.504	0.001
*Festuca rubra*	0.0	3.2	2.2	0.0	**25.4**	0.498	0.002
*Dactylis glomerata*	0.0	0.0	3.2	12.5	**33.1**	0.546	0.001
*Achillea millefolium*	6.7	1.6	2.2	6.9	**43.8**	0.634	0.001
*Kali turgidum*	**13.3**	**14.5**	0.0	6.9	0.0	0.344	0.009
*Polygonum persicaria*	**6.7**	**14.5**	0.0	0.0	6.2	0.304	0.026
*Lepidium ruderale*	**6.7**	4.8	0.0	**13.9**	0.8	0.328	0.009
*Tripleurospermum inodorum*	**33.3**	14.5	0.0	**27.8**	3.1	0.473	0.001
*Rubus caesius*	**13.3**	0.0	2.2	2.8	**13.8**	0.358	0.014
*Senecio jacobaea*	**6.7**	0.0	0.0	0.0	**18.5**	0.415	0.004
*Trifolium arvense*	**20.0**	1.6	0.0	4.2	**20.8**	0.390	0.011
*Trifolium pratense*	**26.7**	1.6	1.1	2.8	**26.2**	0.469	0.002
*Holcus lanatus*	**26.7**	0.0	0.0	0.0	**13.1**	0.381	0.011
*Plantago lanceolata*	**26.7**	4.8	1.1	8.3	**26.9**	0.496	0.002
*Atriplex prostrata*	0.0	**17.7**	0.0	**11.1**	0.8	0.374	0.005
*Poa pratensis*	0.0	8.1	**6.5**	2.8	**22.3**	0.381	0.027
*Urtica dioica*	0.0	1.6	**28.0**	0.0	**14.6**	0.449	0.008
*Deschampsia cespitosa*	6.7	4.8	**33.3**	1.4	**12.3**	0.448	0.006
*Hypochoeris radicata*	0.0	0.0	0.0	**11.1**	**8.5**	0.307	0.035
*Pilosella officinarum*	0.0	1.6	2.2	**13.9**	**14.6**	0.348	0.028
*Hypericum perforatum*	0.0	3.2	3.2	**20.8**	**22.3**	0.442	0.006
*Tanacetum vulgare*	6.7	6.5	0.0	**16.7**	**26.2**	0.462	0.008
*Hieracium piloselloides*	0.0	4.8	6.5	**30.6**	**17.7**	0.410	0.025
*Agrostis capillaris*	0.0	9.7	10.8	**13.9**	**31.5**	0.449	0.016
*Picris hieracioides*	0.0	0.0	3.2	**20.8**	**32.3**	0.527	0.002
*Lotus corniculatus*	13.3	4.8	2.2	**22.2**	**37.7**	0.549	0.001
*Echium vulgare*	0.0	14.5	1.1	**40.3**	**26.9**	0.528	0.002
*Calamagrostis epigejos*	13.3	61.3	9.7	**80.6**	**87.7**	0.892	0.001
*Hordeum jubatum*	**6.7**	**12.9**	0.0	**12.5**	0.0	0.348	0.016
*Tussilago farfara*	**20.0**	**27.4**	1.1	**22.2**	10.8	0.474	0.006
*Oenothera biennis*	**13.3**	**24.2**	0.0	**52.8**	16.2	0.547	0.001
*Polygonum aviculare*	**73.3**	**38.7**	0.0	**34.7**	2.3	0.629	0.001
*Leontodon hispidus*	**6.7**	1.6	0.0	**12.5**	**13.8**	0.355	0.011
*Rumex acetosella*	**6.7**	1.6	0.0	**8.3**	**13.8**	0.334	0.025
*Cirsium vulgare*	**6.7**	6.5	0.0	**9.7**	**13.8**	0.328	0.035
*Trifolium repens*	**20.0**	6.5	1.1	**8.3**	**19.2**	0.380	0.014
*Medicago lupulina*	**20.0**	1.6	0.0	**27.8**	**16.2**	0.448	0.002
*Artemisia vulgaris*	**20.0**	0.0	1.1	**11.1**	**28.5**	0.469	0.003
*Taraxacum officinale*	**26.7**	11.3	7.5	**33.3**	**26.2**	0.506	0.003
*Cirsium arvense*	**26.7**	8.1	2.2	**19.4**	**34.6**	0.497	0.001
*Phragmites australis*	6.7	**21.0**	6.5	**26.4**	**28.5**	0.507	0.001
*Erigeron annuus*	6.7	**16.1**	2.2	**19.4**	**30.8**	0.482	0.003
*Daucus carota*	0.0	**25.8**	4.3	**44.4**	**40.0**	0.604	0.001
*Solidago gigantea*	0.0	14.5	**19.4**	**25.0**	**32.3**	0.499	0.025
*Erigeron canadensis*	**53.3**	**50.0**	2.2	**43.1**	**27.7**	0.615	0.001

**TABLE 4:** Analysis of variance for the community‐weighted mean (CWM) of ecological indicator values (EIV) and functional traits describing studied vegetation among remote sensing‐based clusters, assessed using linear mixed‐effects models (with block nested in heap as random intercepts).

Response	Sum of squares	Mean square	Numerator df	Denominator df	*F*	*p*	Block in heap RE SD	Heap RE SD	Residual RE SD	AIC	AIC_0_	$R_{m}^{2}$	$R_{c}^{2}$
CWM light EIV	27.688	6.922	4	36.46	50.239	<.001	0.327	0.207	0.371	476.7	554.9	.664	.839
CWM moisture EIV	2.670	0.668	4	41.54	1.539	.209	0.354	0.415	0.659	860.2	854.2	.044	.433
CWM soil fertility EIV	7.292	1.823	4	36.79	4.237	.006	0.416	0.475	0.656	873.2	878.5	.142	.555
CWM SLA	112800.843	28200.211	4	42.49	19.752	<.001	17.471	17.108	37.785	3813.6	3886.2	.355	.546
CWM SM	15540.813	3885.203	4	26.30	1.182	.341	18.129	0.003	57.326	4078.1	4101.2	.017	.106
CWM height	100.668	25.167	4	25.41	8.180	<.001	0.603	0.270	1.754	1527.0	1544.4	.125	.234

Abbreviations: AIC, Akaike's Information Criterion; AIC_0_, AIC of null (only intercept and random effects) model; df, degrees of freedom; *F*, test statistic; *p*, *p*‐value; $R_{c}^{2}$, conditional coefficient of determination (proportion of variability explained by both fixed and random effects); $R_{m}^{2}$, marginal coefficient of determination (proportion of variability explained by fixed effects only); RE, random effect; SD, standard deviation; SLA, specific leaf area; SM, seed mass.

### 3.3 Biodiversity

**FIGURE 6:**
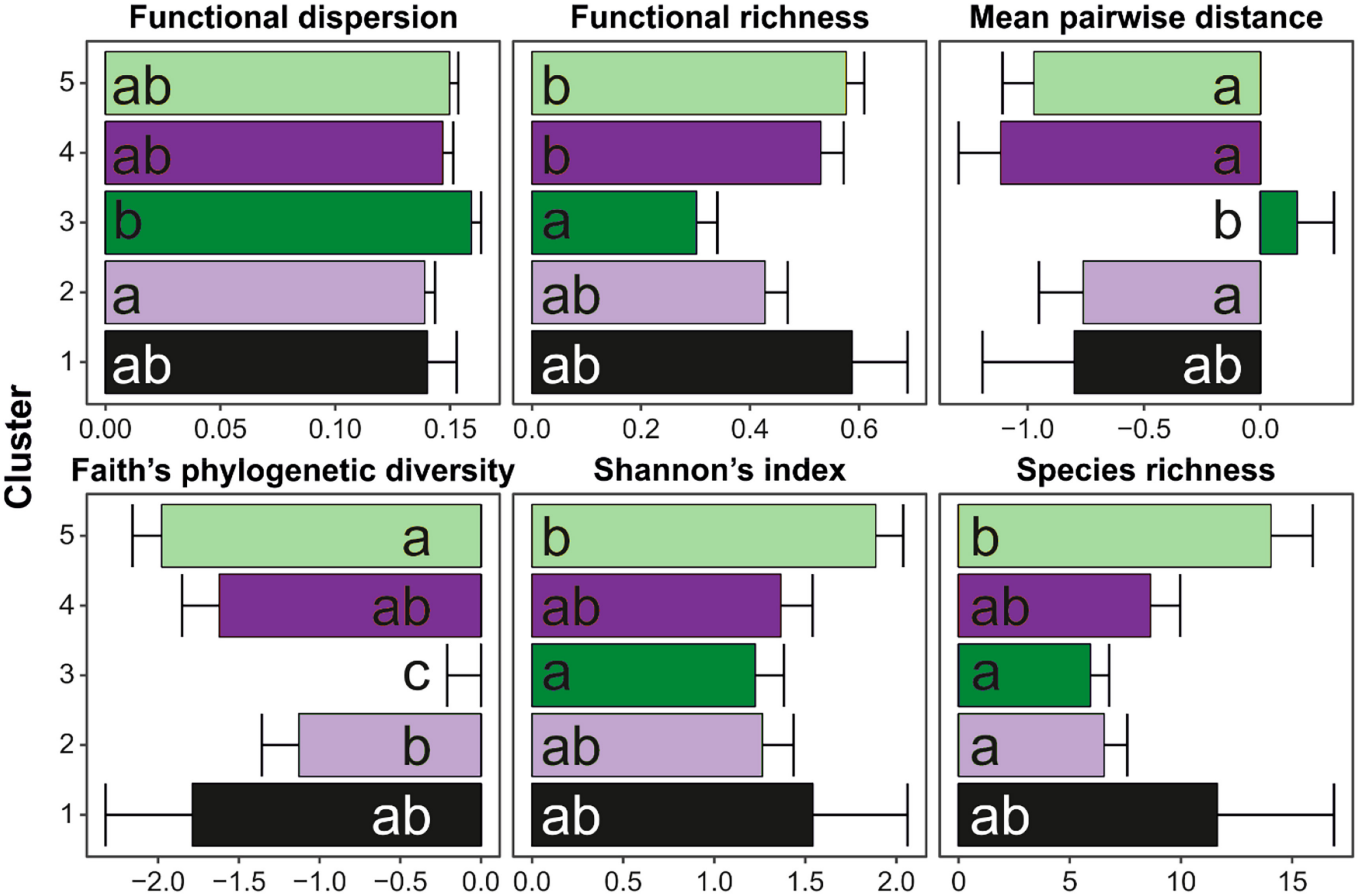
Mean (+SE) values for alpha diversity indices of studied vegetation among remote sensing‐based clusters, assessed using linear mixed‐effects models and a generalized linear mixed‐effect model (Table 5). The same letters denote groups that did not differ at the confidence level *α* = .05 after multiple hypotheses adjustment, according to a Tukey *posteriori* test.

We found the lowest number of species in the 3rd cluster (5.9 ± 8.4 species) while the highest was in the 5th cluster (14.0 ± 1.9 species; Figure 6; Table 5). Shannon's diversity index revealed a similar trend. In contrast, the 3rd cluster had significantly higher phylogenetic diversity, expressed by both Faith's PD and MPD. All other clusters indicated strong phylogenetic clustering while the 3rd cluster revealed random phylogenetic composition (no difference from the null model). The 3rd cluster had the lowest functional richness, twice as low as the 4th, and the 5th cluster had the highest values. However, the 3rd cluster had the highest functional dispersion.

**TABLE 5:** Analysis of variance for alpha diversity indices of studied vegetation among remote sensing‐based clusters, assessed using linear mixed‐effects models (with block nested in heap as random intercepts) and a generalized linear mixed‐effects model with Poisson distribution for species richness.

Response	Sum of squares	Mean square	Numerator df	Denominator df	*F*	*p*	Block in heap RE SD	Heap RE SD	Residual RE SD	AIC	AIC_0_	$R_{m}^{2}$	$R_{c}^{2}$
Faith's PD	55.944	13.986	4	33.93	16.626	<.001	0.532	0.372	0.917	1097.3	1134.1	.329	.552
Mean pairwise distance	26.099	6.525	4	33.24	10.891	<.001	0.449	0.219	0.774	931.4	950.9	.228	.455
Functional richness	1.034	0.259	4	34.47	10.297	<.001	0.092	0.076	0.158	−187.8	−179.7	.242	.516
Functional dispersion	0.006	0.002	4	39.85	3.592	.014	0.002	0.011	0.021	−1722.7	−1751.3	.077	.293
Shannon's index	3.543	0.886	4	41.24	4.242	.006	0.263	0.482	0.457	615.0	618.4	.142	.649
Species richness	33.996	8.499	4	NA	8.499	<.001	0.282	0.414	NA	2194.0	2212.3	.279	.805

Abbreviations: AIC_0_, AIC of null (only intercept and random effects) model; AIC, Akaike's Information Criterion; df, degrees of freedom; *F*, test statistic; PD, phylogenetic diversity; *p*, *p*‐value; $R_{c}^{2}$, conditional coefficient of determination (proportion of variability explained by both fixed and random effects); $R_{m}^{2}$, marginal coefficient of determination (proportion of variability explained by fixed effects only); RE, random effect; SD, standard deviation.

## 4 DISCUSSION

### 4.1 Remotely sensed characteristics of vegetation and indicative species

Remote‐sensing classification of post‐mining areas shows a mosaic of vegetation types due to patchy characteristics of the mineral material of the heaps (Hüttl & Weber, 2001; Kirmer et al., 2008). Axis PC1 was differentiated into vegetated and sparsely vegetated sites. This differentiation reflects the successional development of the vegetation and the remotely sensed clusters which can be combined as clusters 1, 2, and 4—early‐succession, cluster 5—mid‐successional stage, and cluster 3—late‐successional stage. Among remotely sensed characteristics, vegetation height (VH) was divided into meadow and forest suggesting a transition from mid‐ to late‐successional stage. Axis PC2 divided the early successional stage according to thermal‐related indices and proportion of reflectance. This indicates sites that are close to black surface color (i.e., newly established spoil heaps) and areas with sparse vegetation (i.e., pioneer species). Our results could be affected by temporal resolution—we used images acquired in summer when only some of the species flower, which could affect spectral indices based on red, blue, and infrared. However, to minimize that effect we averaged images from the beginning of July with those from September, to capture both flowering and nonflowering dates.

Observed vegetation pattern revealed by ordination (NMDS) showed ecological interpretation of clusters—the development of plant species from the early successional stage (1st, 2nd, and 4th clusters) through mid‐succession (5th cluster) to late succession (3rd cluster). We found 15 indicative species (i.e., 13 in the 1st cluster and one each in the 2nd and 4th clusters) in early succession. The indicative grassland species *Lolium perenne* showed significantly higher frequency in early successional stages. In a 6‐year permanent plot study, Alday et al. (2011b), found that cover of *Lolium perenne* declined over time during early succession on coal wastes in northern Spain. Other indicative species, including *Oxybasis* spp., *Chenopodium album*, and *Echinochloa crus‐galli*, with ruderal characteristics (Klotz et al., 2002) were highly frequent in early succession. This is expected considering the openness of spoil heaps during the early stages of vegetation development. In a similar study in Upper Silesia, a high frequency of ruderal species was found on young spoil heaps (Piekarska‐Stachowiak et al., 2014).

The mid‐successional stage (i.e., 5th cluster) differed by having a high frequency of indicative species characterized by dense low vegetation (i.e., meadow and grassland): *Achillea millefolium*, *Dactylis glomerata*, *Festuca rubra*, *Galium mollugo*, *Silene latifolia*, and *Vicia cracca*. Prach (2013), referred to the mid‐succession stage as *post‐ruderal*—the period where ruderal species are substituted by nonruderals. Frouz et al. (2008) showed that the mid‐successional stage corresponds to a period of substantial change in soil structure and biota.

In the late‐successional stage (i.e., 3rd cluster), we found a high frequency of ancient forest indicator species (Hermy et al., 1999): *Lolium giganteum*, *Circaea lutetiana*, *Millium effusum*, and *Poa nemoralis*, as well as forest‐edge species: *Rubus idaeus*, *Geum urbanum*, and *Impatiens parviflora*. Jabs‐Sobocińska et al. (2022) found *Circaea lutetiana* among the species significantly occurring more often in recent forests in the Carpathian. Although shade‐tolerant *Millium effusum* is generally considered an ancient woodland indicator (De Frenne et al., 2017), it is also known to colonize secondary, post‐agricultural forests in Poland (Brunet et al., 2012) and Sweden (Brunet et al., 2012). *Poa nemoralis* is a moderately strong indicator of ancient forest. When not hindered by dispersal limitation and elevated nutrient levels, *P. nemoralis* rapidly colonizes recently established forest areas adjacent to ancient forests (Plue et al., 2020). That way the presence of such forest species indicates the progress of secondary succession, the dynamics of which was reflected in vegetation clusters determined in our study based on remote sensing.

### 4.2 Change in species richness and diversity along a successional gradient

Species diversity in post‐mining areas is controlled by mechanisms of community assembly which represents our remotely sensed cluster classification. There are two main models explaining how species diversity responds to community succession. The first posits that diversity increases throughout succession by migration and decreases over time through competition (Odum, 1969). The second suggests that diversity gradually increases at early succession when pioneers dominate, becomes at maximum in mid stages when there are still pioneers but the mid and late successional species are already beginning to establish, and diversity gradually falls into the late stages of succession when the pioneers are eliminated, revealing an arch‐shaped pattern (Connell, 1978). In this study, we discovered that both species richness and Shannon diversity had a clear trend as succession progressed. While species richness and Shannon diversity were significantly higher in the early and mid‐successional stages than in late succession, the mid‐successional stage became the maximum in these response variables (an arch‐shape pattern). Therefore, our study showed that remotely sensed vegetation clusters can help with ground data collection to reveal an arch‐shaped pattern of species richness and Shannon diversity as proposed by Connell (1978). Reduced competition among herbaceous and shrub species in mid‐succession provides for the establishment of species such as *Senecio jacobaea*, *Trifolium arvense*, *Holcus lanatus*, *Hypochoeris radicata*, *Tanacetum vulgare*, *Leontodon hispidus*, *Rumex acetosella*, and *Cirsium vulga*re that are indicative of early and mid‐successional species. Similarly, light availability (Bazzaz, 1979) and surrounding vegetation near spoil heaps (source of propagules) may support the establishment of more species through seed dispersal in the mid‐successional stage (Czortek, 2023; Prach & Rehounková, 2006). On the other hand, competition among species for limited resources, particularly light availability due to canopy closure, can be the main cause of the decline in species diversity in the late successional stage (Prach et al., 2013). This trend was confirmed by Rawlik et al. (2018) who found that similar age stands of tree species transmitting more light through their canopies supported the presence of more understory species and higher biomass. Our results are in agreement with other studies (Alday, Pallavicini, et al., 2011; Shafi & Yarranton, 1973; Wiegleb & Felinks, 2001a). However, in contrast to our findings, a linear increase in species richness and diversity with spoil heap age was documented in some studies (Hazarika et al., 2006; Piekarska‐Stachowiak et al., 2014; Pietrzykowski, 2008). Other authors found the highest diversity in the early and late stages of succession, representing a U‐shaped pattern (Badraghi et al., 2023; Hilmers et al., 2018). While our study and that of Piekarska‐Stachowiak et al. (2014) were conducted in the same region, it should noted that Piekarska‐Stachowiak et al. (2014) used the most frequent dominant species groups in the vegetation to establish permanent plots instead of random allocation of sample plots to vegetation patches. This might be an important driver of the difference in results, as preferential sampling affects the results of vegetation analyses (Holeksa & Woźniak, 2005). Therefore, our approach of random allocation of sample plots to vegetation patches helps remove bias and provides robust findings. In general, a nearly universal pattern of diversity could be expected—the diversity of the late stage must be lower than that of some preceding stages unless the late stage is affected by disturbance allowing the establishment of pioneers in the late‐successional stage (Horn, 1974).

### 4.3 Changes in functional and phylogenetic strategies along a successional gradient

Our study revealed differences in functional diversity between obtained remote‐sensed clusters which follow successional development. We found a significant difference in community‐weighted means (CWM) of ecological indicator values (EIV) and functional traits (light EIV, soil fertility EIV, height, and SLA) between clusters indicating the increasing importance of competition as succession advances. The significant CWM of light EIV in late succession (i.e., 3rd cluster) suggests the influence of abiotic filtering as plant species in this stage show low demand for light during the juvenile stage; thus the elimination of plant species that perform best at optimum light and in open habitat. Similarly, through low photosynthetic rates, the late‐successional species are usually more efficient at low light availability (Bazzaz, 1979). The difference in CWM soil fertility EIV in late succession (i.e., 3rd cluster) suggests an increasing need for soil nutrients as succession progresses. Similarly, plant height was significantly higher in late succession (i.e., 3rd cluster) which can infer that plant species at late succession are highly productive with strong competitive capacity.

SLA is an important trait that explains an acquisitive‐conservative trade‐off (Wright et al., 2004), which differed between our obtained remotely sensed clusters. We expect a decrease in SLA along a successional gradient (e.g., Boukili & Chazdon, 2017; Lohbeck et al., 2013) due to its positive relationship with relative growth rate (Wright et al., 2004). However, in our study, the CWM of SLA was significantly higher in late succession (i.e., 3rd cluster). SLA variation is influenced by two traits: leaf dry matter content (LDMC) and leaf thickness (Hodgson et al., 2011; Witkowski & Lamont, 1991). While leaf thickness is positively related to light availability, LDMC is negatively related to soil fertility (Hodgson et al., 2011). Thus, decreased thickness with shading in late succession indicates a reduction in the division and expansion of palisade chlorenchyma cells (Dengler, 1980) to minimize internal shading of chloroplasts. Therefore, SLA can increase due to increased shade and soil fertility. In general, the differences in traits can be explained by variations in light and nutrient availability, both at interspecific and intraspecific levels (Grime, 2006; Paź‐Dyderska et al., 2020; Poorter et al., 2005).

Functional diversity differed between obtained remotely sensed clusters. We found the lowest functional richness and highest functional dispersion in the late successional stage (i.e., 3rd cluster). Low functional richness may indicate high environmental filtering (Laliberté & Legendre, 2010), as it estimates the amount of niche space filled by all species in a community. Therefore, if plant community composition is constrained by environmental filtering, the range of available niches should be limited and we can expect a low functional richness. Similarly, shade is an important environmental filter supporting a few unique species in the late‐successional stage (i.e., 3rd cluster), thus, the low functional richness. In our study, the most frequent species in late succession—*Lolium giganteum*, *Circaea lutetiana*, *Millium effusum*, *Poa nemoralis*, *Geum urbanum*, and *Impatiens parviflora* are adapted to shade or moderate shade conditions. Functional dispersion (FDis) measures the mean distance of all plant species to the weighted centroid of the community in trait space (Anderson et al., 2006). A high value of FDis is an indication of low habitat filtering. FDis is independent of species richness but takes into account species abundance (Laliberté & Legendre, 2010). We recorded a high cover of species with distinct traits typical of forest ecosystems, for example, *Circaea lutetiana*, *Poa nemoralis*, *Sambucus nigra*, *Milium effusum*, *Impatiens parviflora*, and *Geum urbanum* in the late‐successional stage (i.e., 3rd cluster) which could cause FDis to be high. The high cover of these species in comparison to the total species pool especially since these dominant cover species are adapted to shade conditions in late succession, resulted in that trend.

Phylogenetic diversity reflected the differences between our remotely sensed clusters. The late‐successional stage (i.e., 3rd cluster) had significantly higher phylogenetic diversity (Faith's Phylogenetic diversity and Main pairwise distance). Given that phylogenetic randomness and clustering are explained by environmental filtering (Emerson & Gillespie, 2008), under this hypothesis, abiotic conditions (i.e., temperature, precipitation, soil nutrients, and sunlight) filter species with similar trait combinations (Keddy, 1992). We could infer that environmental filters such as light and soil nutrients account for the phylogenetic nonrandomness of late‐successional plant communities. Plant communities in the herbaceous layers are adapted to shade conditions while those in the shrub, understory, and canopy layer compete for sunlight. Similarly, competitive exclusion controls the plant communities in late succession because species compete for resources. Competitive exclusion can filter shade‐intolerant species, thus contributing to phylogenetic convergence in late succession. Also, our findings suggest that competitive exclusion determines the species composition during the late stages of succession because environmental conditions such as light are highly heterogeneous (i.e., not homogeneously distributed) (Matsuo et al., 2021). In addition, in the late‐successional stage, most light is intercepted at higher strata of vegetation, decreasing the amount of light beneath the canopy, and excluding light‐demanding species from the community. As a result of the competitive effect, we have distinct plant species at the canopy layer, shrub layer, and herb layer, making the phylogenetic diversity at a similar level than predicted under a null model in late succession.

## 5 CONCLUSIONS

Our study demonstrated that objective a priori classification of vegetation using remotely sensed data can help elucidate meaningful and ecologically interpretable division. Using the chronosequence of spontaneously developed vegetation in post‐mining sites, we confirmed the applicability of remote sensing for designating study sites suitable for ecological studies. Remotely sensed characteristics differentiated early‐, mid‐, and late‐successional stages. Species composition revealed that early‐successional stages hosted species indicative of grassland and ruderal species, mid‐successional stages had the highest proportion of meadow species, while late‐successional stages were characterized by the presence of forest and forest edge species. These stages were reflected in remote sensing‐divided clusters of study sites. Species richness and diversity followed an arch‐shaped pattern: they were the highest in mid‐succession and lowest in late succession. Functional composition differed significantly in late succession for light EIV, soil fertility EIV, CWM for plant height, and SLA. The late succession vegetation had the lowest functional richness and highest functional dispersion. We also found a difference in phylogenetic diversity. All these trends were in line with previous ground‐based studies, showing that remote sensing can help with the objective and low‐cost selection of study sites for the assessment of vegetation restoration success. That way it can provide new insights into ecosystem diversity between vegetation types along successional gradients in post‐mining heaps.

## AUTHOR CONTRIBUTIONS

**Quadri A. Anibaba:** Conceptualization (lead); data curation (lead); formal analysis (equal); methodology (lead); project administration (supporting); visualization (equal); writing – original draft (lead); writing – review and editing (lead). **Marcin K. Dyderski:** Conceptualization (equal); methodology (equal); writing – review and editing (supporting). **Gabriela Woźniak:** Conceptualization (supporting); investigation (supporting); methodology (supporting); writing – review and editing (supporting). **Andrzej M. Jagodziński:** Conceptualization (supporting); funding acquisition (lead); writing – review and editing (supporting).

## CONFLICT OF INTEREST STATEMENT

We declare that there are no known competing financial interests or personal relationships that could have appeared to influence the work reported in this paper.

## Supporting information


Data S1.

## Data Availability

The data for this study are accessible on the FigShare repository: https://doi.org/10.6084/m9.figshare.25289401.
