# Supplementary figures and images for: Remote sensing for site selection in vegetation survey along a successional gradient in post‐industrial vegetation
Source: Ecol Evol. 2024 Aug 27;14(8):e70200. doi: 10.1002/ece3.70200 (PMC11349488; doi:10.1002/ece3.70200)

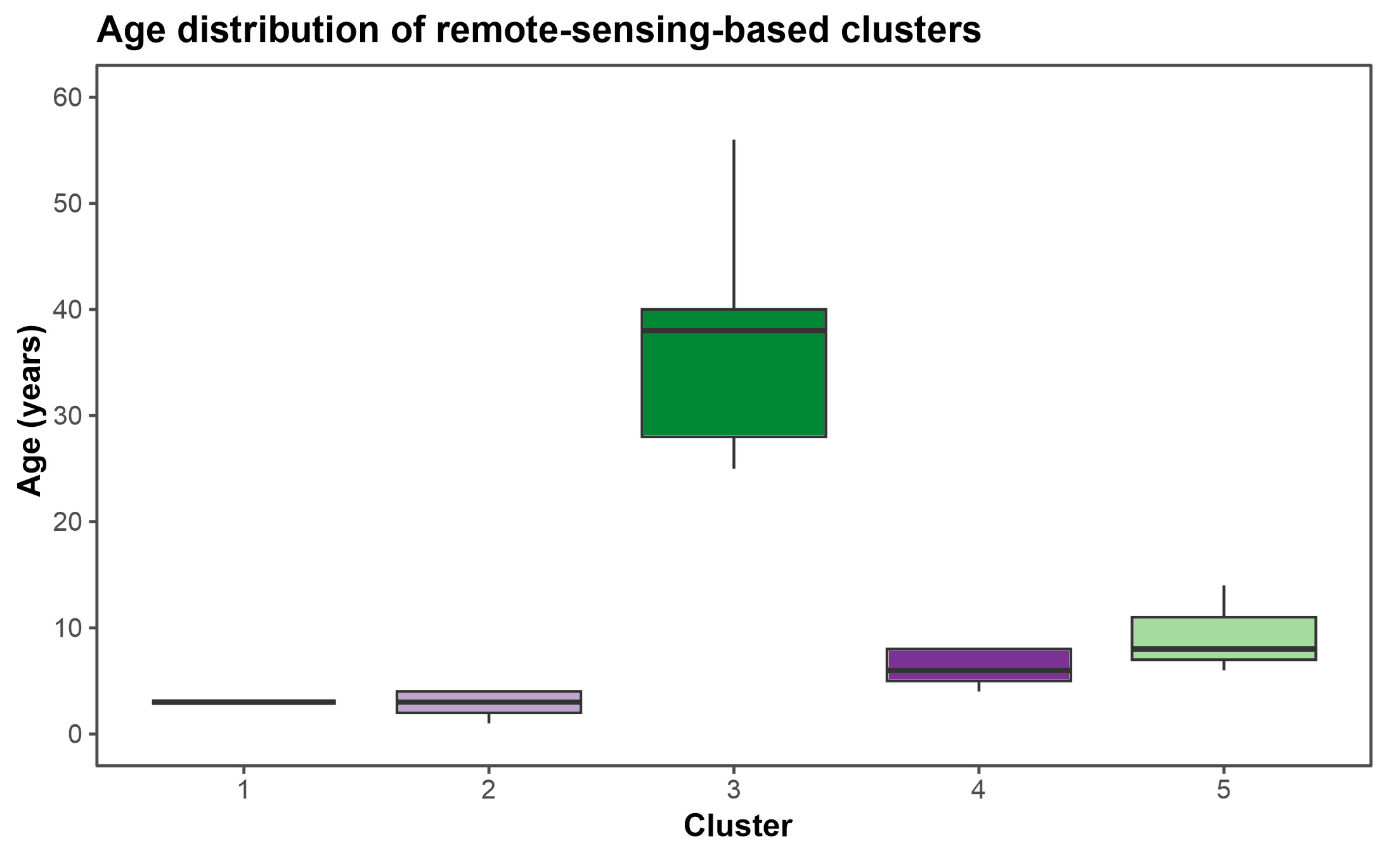


**Figure 1**: Age distribution of remote-sensing-based clusters

Supplement: Supplementary file 1 — Data S1. [file ECE3-14-e70200-s001.docx]
